# A Complex Intervention to Minimize Medication Error by Nurses in Intensive Care: A Case Study

**DOI:** 10.3390/healthcare13010066

**Published:** 2025-01-02

**Authors:** Fábio Coelho, Luís Furtado, Márcio Tavares, Joana Pereira Sousa

**Affiliations:** 1 Department of Nursing, Mental Health, and Gerontology, School of Health, University of the Azores, 9700-042 Angra do Heroísmo, Portugal; luis.cr.furtado@uac.pt; 2 Department of Nursing, Family and Community, School of Health, University of the Azores, 9500-321 Ponta Delgada, Portugal; marcio.fm.tavares@uac.pt; 3 School of Health Sciences, Polytechnic of Leiria, Rua General Norton de Matos, Apartado 4133, 2411-901 Leiria, Portugal; joana.sousa@ipleiria.pt; 4 Center for Innovative Care and Health Technology (ciTechCare), 2411-090 Leiria, Portugal; 5 Nursing Research, Innovation and Development Centre of Lisbon (CIDNUR), Nursing School of Lisbon, 1600-190 Lisbon, Portugal

**Keywords:** nurses, medication errors, intensive care units, case study, focal group

## Abstract

**Background/Objectives:** Medication errors are the most frequent and critical issues in healthcare settings, often leading to worsened clinical outcomes, increased treatment costs, extended hospital stays, and heightened mortality and morbidity rates. These errors are particularly prevalent in intensive care units (ICUs), where the complexity and critical nature of the care elevate the risks. Nurses play a pivotal role in preventing medication errors and require strategies and methods to enhance patient safety. This study aims to develop a comprehensive and evidence-based intervention to minimize medication errors by nurses in ICUs. **Methods:** This qualitative case study forms a part of a broader research project that commenced with a scoping review. Building on the review findings, a complex intervention was designed to address nurses’ medication errors. A focus group of experts was conducted to validate the intervention designed, evaluating its contextual feasibility and relevance. **Results:** This study led to the development of a complex intervention whose relevance lies in its potential implementation within the studied context. The resulting intervention was structured around four main components—educational interventions, verification and safety methods, organizational and functional modifications, and an error reporting system—meticulously designed to leverage the ICU’s existing resources. **Conclusions:** In conclusion, the proposed intervention has the potential to positively impact healthcare quality by reducing errors and promoting a culture of safety. Furthermore, this study’s findings provide a relevant foundation for future research and practical applications, driving advancements in healthcare service excellence.

## 1. Introduction

Medication errors are a significant global concern, posing severe risks to patients, healthcare professionals, and systems [1]. The increasing use of medications, driven by advancements in medicine, heightens the potential for errors, leading to adverse outcomes for patients, families, and healthcare systems [1].

A medication error is defined as any preventable event that may result in inappropriate medication use or patient harm at any stage of the medication management process [2].

This process—spanning selection, acquisition, storage, prescription, verification, preparation, dispensing, administration, and monitoring—is inherently complex and vulnerable to errors [3,4].

Research shows that approximately one-third of harmful medication errors occur during the preparation and administration stages, with nurses playing a pivotal role in these phases [4]. Positioned at the end of the medication management chain, nurses are critical in preventing medication errors and ensuring patient safety [5,6,7]. Their responsibilities encompass not only preparing and administering medication, but also fostering a safe care environment through corrective measures and error-prevention strategies within a non-punitive system [7].

Medication administration errors by nurses are the most frequently reported worldwide [8]. However, the actual prevalence of such errors is challenging to quantify due to underreporting, often driven by fear of retaliation [1]. In the United States alone, approximately 400,000 medication errors are reported annually, contributing to at least one daily death and affecting over 1.3 million individuals annually [9,10]. Globally, these errors account for nearly 1% of healthcare expenditures, amounting to an estimated $42 billion [10].

Reported error rates vary widely across countries, with Germany and England documenting rates of 4.78% and 3.22%, respectively, while Brazil reports significantly higher rates, with 64.3% of errors occurring predominantly during medication preparation and administration [5]. A study in a Brazilian university hospital identified 16,753 medication errors between 2007 and 2013, 18.9% of which were linked to adult intensive care units (ICUs) [11].

ICUs present an elevated risk for medication errors due to the critical condition of the patients, the intensive use of intravenous medications, and the highly technical environment [12]. It is estimated that ICU patients experience an average of 1.7 errors per day of hospitalization, with 8% to 10% of ICU patients falling victim to incidents related to medication management practices [3,13].

Given the severe consequences and economic burden associated with medication errors, safety within the medication management process requires urgent attention. These errors are a key quality indicator for patient care [1,4,14]. Nurses, as central players in this process, require targeted guidance to identify, notify, and prevent errors effectively [6,15]. Implementing preventive measures is essential to enhance patient safety and healthcare quality [15].

Nursing has made strides in implementing strategies to improve workflow and enhance professional reliability. However, developing and evaluating health interventions that improve patient outcomes and system effectiveness remains complex yet essential, particularly in nursing, where interventions often involve multifaceted processes [16]. This study, therefore, aims to develop a comprehensive intervention to promote good practices and ensure safety in minimizing medication errors by nurses in intensive care settings.

## 2. Methods

**Figure 1:**
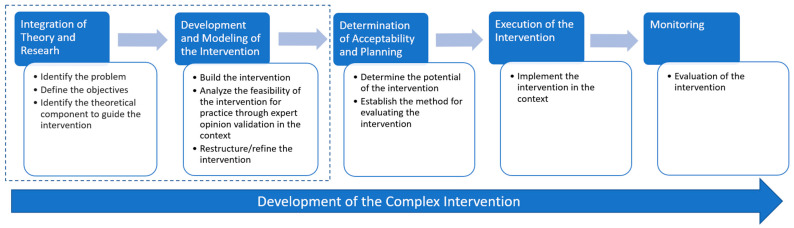
A flowchart representing the phases of the complex intervention development process.

This study employs a qualitative case study method and is a part of a broader research project. The larger study included the following: (1) a scoping review, already published [17,18], (2) the present manuscript, which pertains to the development of the intervention, (3) its implementation, and (4) the evaluation of its impact (Figure 1).

The scoping review [18] aimed to identify interventions and strategies to reduce medication errors by nurses, supporting the development of a safety-promoting intervention. The review also sought to map the factors influencing medication errors and examine their consequences within adult intensive care unit settings.

Building on scoping the review’s findings, a complex intervention was designed to address medication errors among nurses. The intervention was then validated through a focus group, which assessed its suitability and feasibility within the targeted context.

### 2.1. Participants Selection

The study was conducted in a hospital located on an island within Portuguese territory. The hospital has a total capacity of 313 beds, 210 of which are allocated to general wards. The intensive care unit under study comprises 8 beds [19].

A purposive sampling approach [18] was adopted, selecting participants who could best represent the phenomenon under investigation. Eight ICU nurses meeting the inclusion criteria were included in the focus group. The inclusion criteria were as follows: (a) registered nurses specializing in Medical-Surgical Nursing; (b) active employment in an intensive care unit; (c) minimum of five years of professional experience in intensive care settings; and (e) engagement in direct patient care exclusively.

The exclusion criteria were as follows: nurse managers and general care nurses.

### 2.2. Data Collection and Focus Group

The focus group was conducted via Zoom^®^ platform (Zoom Video Communications, Inc., San Jose, CA, USA), facilitating the participation of geographically dispersed experts. Participants were recruited through phone calls and a closed questionnaire on WhatsApp^®^ (Meta Platforms, Inc., Menlo Park, CA, USA) to confirm availability. This was followed by a formal email invitation providing the session details, including date, time, and the Zoom access link.

An interview guide, informed by the scoping review, served as the data collection instrument (Appendix A). The guide prompted participants to provide reasoned opinions on the intervention’s strategies.

As this study relied on consensus-building, a content analysis approach [20] was employed to evaluate the experts’ feedback, providing justification for including or excluding individual strategies within the intervention.

### 2.3. Data Treatment and Analysis

The focus group discussion was transcribed and subjected to content analysis [21]. An a priori categorization framework was developed based on the intervention’s components (categories) and plans (subcategories) derived from the scoping review. This framework was used to validate the intervention’s contextual appropriateness and feasibility.

### 2.4. Ethical Considerations

The study adhered to the ethical standards outlined in the Declaration of Helsinki, the World Health Organization, and the European Union’s regulations for research involving human participants. It also complied with the provisions of Law No. 21/2014 governing clinical research in Portugal.

Participation was voluntary, with no associated risks or costs to participants. Confidentiality and anonymity were ensured, with participants receiving detailed information about the study and signing informed consent forms. Additional safeguards were employed for focus group confidentiality, including a disclaimer obligating the researcher to maintain participants’ anonymity and protect all shared information.

Data from the focus group were securely stored on the principal investigator’s computer for six months before deletion. The researcher transcribed the discussion to ensure data confidentiality. Ethical approval for the study was granted by the Hospital de Santo Espírito da Ilha Terceira Ethics Committee (approval number 11/2023).

### 2.5. Complex Intervention Design

Complex nursing interventions can address various aspects of care, including multidisciplinary team collaboration, patient education and counseling, behavioral strategies, care coordination, technological innovations, supportive care services, quality improvement initiatives, and patient-centered care planning [22]. The intervention developed in this study falls under the domain of quality improvement initiatives, as it represents an evidence-based approach to enhancing nurses’ performance and improving the safety and quality of nursing care.

Developing complex interventions requires a rigorous methodological framework to ensure all necessary components are effectively integrated [23]. The active engagement of stakeholders is essential for ensuring the intervention addresses the actual needs of patients and healthcare professionals. Furthermore, adherence to established guidelines, such as those provided by the Medical Research Council (MRC), is crucial for designing interventions based on robust evidence, thus optimizing outcomes [23]. The updated MRC guidelines also provide valuable insights for advancing nursing knowledge, enabling the development of practices that benefit patients and improve the overall care delivery [16,24].

The first step in creating a complex intervention is to establish a strong evidence base [24]. Following fundamental conceptual principles is vital for maximizing the likelihood of achieving the intended outcomes. This study began with a scoping review [18] that identified effective interventions for preventing medication errors by intensive care nurses, examined risk factors, and provided a foundation for constructing the intervention.

Building on the findings of the literature review, a careful and systematic analysis was conducted to design strategies that effectively address the issue of medication errors. The resulting intervention model focuses on minimizing medication errors in intensive care units and requires validation to account for the specific organizational and functional characteristics of individual ICUs. Recognizing the diverse factors influencing organizational dynamics, the intervention takes a holistic approach to foster sustainable and effective change.

Personal factors, such as fatigue, distraction, and interpersonal conflicts, were not directly included in the intervention. Instead, these factors are expected to improve indirectly through the implementation of strategies that address broader organizational challenges.

The intervention is structured around the implementation of safe practices and the mitigation of factors contributing to medication errors among nurses. It consists of four key components, each supported by specific plans and activities to achieve the desired outcomes. Table 1 outlines the detailed framework of the complex intervention, derived from the scoping review.

The designed intervention resulted from the contributions from the scoping review, which the focus group participants evaluated. With in-depth knowledge of the clinical practice context, these participants critically assessed the suitability of the various components and intervention plans, eliminating some and adapting others.

The effectiveness of a complex is highly dependent on the context in which it is applied [25,26]. An intervention successful in one setting may not yield similar results elsewhere [25]. Therefore, it is imperative to assess its adaptability to different environments. In this study, the intervention’s suitability and feasibility were validated through a focus group, emphasizing the importance of tailoring interventions to the unique challenges and requirements of specific ICU settings. Such contextual adaptation is especially critical in environments as intricate and sensitive as intensive care units.

## 3. Results

The focus group participants analyzed and discussed the previously constructed complex intervention aimed at reducing medication errors by nurses in an ICU. This session brought together nurses specializing in Medical-Surgical Nursing who shared their experiences, perceptions, and suggestions regarding the intervention’s design and its impact on clinical practice. The transcription and categorization of the data provided valuable insights into the intervention’s feasibility and allowed adjustments to ensure its suitability for the specific ICU context under study.

Out of eight nurses who met the inclusion criteria, six participated in the focus group (Table 2). Despite this, the panel’s composition complied with the number of participants recommended in the literature [27,28].

From the categorization process, four categories have emerged as follows: (1) educational intervention, (2) verification and safety methods, (3) organizational and functional modifications, and (4) error reporting system. The categorization process is illustrated in Figure 1, which aids in understanding the intervention’s structure.

### 3.1. Educational Intervention

Participants emphasized the importance of creating discussion groups to promote safety in the medication process and define intervention plans based on monitored needs. As one participant noted, *“This moment of discussion should be a starting point for defining an intervention plan, but without being systematic or organized”* (P3). Another added, *“There should be constant monitoring of needs so that we’re not holding meetings without a very concrete objective”* (P1).

The group highlighted the need to include less experienced nurses in these discussions to facilitate their integration and address specific challenges. As one participant suggested, *“It would make more sense to have a collective discussion with the team about the difficulties they have experienced, particularly with nurses with less time on the job”* (P3). Another agreed, stating, *“It makes sense to create discussion groups with nurses who have only been working in the intensive care unit for a short time”* (P2).

Participants also stressed the usefulness of checklists, posters, and videos to standardize and disseminate critical information. They cited examples such as, *“We have a list with the dilutions of the medication and how to prepare it, which is an excellent strategy”* (P4) and *“In our reality, we have a good tool, which is the information system that, on the labels available, gives the dilution of the medication”* (P5).

The use of slide presentations emerges as a versatile strategy for sharing data derived from the monitoring of indicators related to the medication management process and for addressing topics in pharmacology. As highlighted by one expert, this approach could involve *“a working group where the structure, process, and outcome indicators are identified, (…) followed by a sharing session after monitoring the entire medication cycle”* (P3). They further emphasized that *“the use of PowerPoint to address issues associated with pharmacology also seems like a measure to be implemented”* (P3).

The potential for using videos as an educational strategy was also emphasized. One participant suggested, *“We should film a moment of medication preparation during high workloads for later analysis”* (P3).

Simulated practice sessions were identified as beneficial for less experienced nurses to develop and refine their skills. However, participants advised against making these sessions routine. As one participant commented, *“Promoting simulated practice for younger colleagues is a good strategy, but it shouldn’t be something done systematically”* (P4).

### 3.2. Verification and Safety Methods

Participants strongly advocated for developing and using protocols and operational instructions for specific medications. One participant remarked, *“There is a need for protocols”* (P1), while another elaborated, *“Protocols and operational instructions make sense for certain specific medications”* (P2).

Checklists were seen as valuable, particularly during ICU integration. However, participants recommended general checklists rather than creating one for every medication. As one explained, *“There should be a procedure where nurses, in their basic and advanced integration in the ICU, know the document with indications on preparation and administration. But a checklist for each medication doesn’t seem to make sense”* (P3).

Participants emphasized the importance of monitoring laboratory values before medication administration and assessing vital signs both before and after administration as integral practices for ensuring patient safety. These practices were considered closely related, fitting within the same subcategory. One participant suggested, *“I would put a bar here and put vital parameters”* (P3). The group highlighted specific practices already implemented by nurses, including the identification of relevant laboratory values, and the confirmation of laboratory results and vital signs while considering therapeutic targets, and assessing the patient’s clinical condition. Contacting the prescriber in cases of uncertainty was also noted as a standard approach. Participants provided examples of these practices: *“there are some drugs where the prescription is made for a target value, to be reached or not exceeded, whose values must be taken into account before administration (…). We already do this”* (P1), *“when there is any doubt we ask”* (P5), and *“apart from emergency drugs, all the others we have time to check all the parameters in detail”* (P6).

Reducing interruptions during medication preparation was deemed critical. Participants identified the current ICU environment as highly distracting. One noted, *“This is not possible with the model we currently have. It is an open unit, there are a lot of alarms ringing, and therefore anything can be distracting”* (P3).

Proposed solutions included establishing designated medication preparation areas and using visual identifiers, such as vests, to signal when nurses are preparing medications. As one participant suggested, *“Create a marking that represents a medication preparation area so that when the person is in that space, they can’t be interrupted”* (P1). Another added, *“Wear a vest with the written indication: medication preparation”* (P1).

While participants acknowledged the importance of double-checking high-risk medications, they deemed it impractical due to staffing shortages. As one participant noted, *“I don’t think we have to limit ourselves to two nurses when they are often not available”* (P3). Another added, *“Sometimes there are doubts when preparing a medication, and we turn to someone more experienced to validate it”* (P5).

### 3.3. Organizational and Functional Changes

The participants strongly emphasized the need for structural and organizational changes to support safer medication practices. One of the most frequently discussed aspects was the differentiation of medication containers. High-risk and look-alike medications were identified as critical points of vulnerability. One participant suggested, *“We need specific labels that alert nurses to the similarity and criticality of the medicine”* (P1). Another added, *“This differentiation, combined with focused and attentive nurses, will significantly reduce errors”* (P6). Despite this recognition, the group acknowledged that implementing this strategy would require significant effort and planning. As one participant commented, *“Although it hasn’t been implemented yet, with a little effort, we’ll get there”* (P1).

Participants also proposed improvements in medication storage practices. They emphasized that storing medications with similar labels or appearances in separate locations would reduce the risk of mix-ups. One nurse stated, *“Critical medications should be stored in yellow drawers or boxes to make them stand out”* (P1). However, participants noted that the lack of advanced dispensing systems, such as Pyxis^®^, limited their ability to fully implement individualized storage systems. One participant expressed this frustration, saying, *“If we had a system like Pyxis, where everything was individualized, that would be ideal. Unfortunately, we’re far from that reality”* (P3).

Electronic systems were identified as another area for potential improvement. While electronic documentation systems were already in use, participants recommended introducing color-coded labels for high-risk medications to further enhance safety. One nurse highlighted the need for this by stating, *“I think it would be beneficial if labels could be printed in a different color for high-risk medication”* (P5). They also emphasized that eliminating transcription steps would reduce errors significantly, as transcription often leads to misinterpretation. As one participant explained, *“Anything that involves transcription from a paper system increases the risk of error, so we need to minimize it as much as possible”* (P1).

### 3.4. Error Notification System

The error notification system was recognized as an essential tool for identifying and addressing medication errors. However, participants unanimously agreed that a culture of reporting was lacking in the ICU. Despite having a system in place, it was underutilized due to fear of repercussions and skepticism about anonymity. One participant noted, *“Although the system exists, it isn’t used regularly because people fear they’ll be identified”* (P6). Another added, *“No matter how much we reinforce that the notification is anonymous, people still don’t believe it”* (P3).

Participants acknowledged the potential of the system to drive improvements in clinical practice if used appropriately. One nurse highlighted this by saying, *“Identifying errors through this system would allow us to implement new strategies to reduce them, but we can’t do that if people don’t use it”* (P6). Training on the use of the system was seen as a critical step to improve understanding and confidence in its use. As one participant suggested, *“We need practical training to help nurses navigate the system and understand its importance”* (P1).

Another significant issue was the lack of feedback following error notifications. Participants emphasized that creating and sharing reports derived from the notification process could help close the feedback loop and promote a culture of improvement. One nurse remarked, *“If reports from the notification system were shared with staff in feedback sessions, it would show how the data is being used to improve practice”* (P3). However, this step was considered premature, as the primary focus needed to be on increasing the frequency of error notifications.

### 3.5. Consensus on the Intervention to Be Implemented

The focus group discussions were instrumental in identifying the strengths and weaknesses of the initial intervention and refining it to suit the ICU’s context. While the four main components—educational intervention, verification and safety methods, organizational and functional changes, and error notification system—were retained, several subcategories were either excluded or adjusted based on the feedback.

Certain strategies, such as double-checking medication preparation and monitoring laboratory values or vital signs, were excluded because these practices were already well established in the ICU. As one participant explained, *“We already check laboratory values and vital signs before administering medication, so including this in the intervention wouldn’t add value”* (P1). Another agreed, stating, *“Double-checking is a great idea, but with the current staff shortages, it’s just not feasible to implement it consistently”* (P3).

On the other hand, the use of video as an educational tool was added to the intervention. Participants highlighted its potential to enhance learning by allowing nurses to observe and reflect on their behaviors. One nurse described this as, *“A method of learning about our actions and improving our practice through self-analysis”* (P4). Practical training on the error reporting process was also included, as participants noted that despite previous training sessions, there was still significant confusion about how to use the system effectively.

The revised intervention also emphasized the importance of an ongoing promotion of the notification system and the creation of reports to track progress and identify additional improvement strategies. As one participant put it, *“If we don’t promote the system and show people the results of their notifications, we won’t see a change in reporting behavior”* (P6).

Ultimately, the focus group validated the intervention’s core components while refining specific strategies to ensure their feasibility and relevance. The revised intervention is presented in Table 3, which details the updated components and associated activities.

## 4. Discussion

The focus group provided critical insights into the implementation of strategies for reducing medication errors by nurses in ICUs. Participants emphasized the need to tailor certain strategies to less experienced nurses, including discussion moments on medication safety, simulated practice, the use of checklists, and double-checking during medication preparation. This aligns with studies that have found a correlation between years of nursing experience and correct medication practices [29,30]. However, less experienced nurses are sometimes more meticulous, as they tend to adhere strictly to protocols compared to their more experienced counterparts, who may rely on routine practices and thus be less thorough [31].

Checklists emerged as a pivotal tool for improving care quality and safety. They serve as critical aids to human memory, standardizing complex processes and minimizing errors [32]. Studies emphasize that the validation and implementation of checklists in both clinical and simulated settings can facilitate the early adoption of safe practices [33]. However, for sustained effectiveness, ongoing education and periodic reinforcement sessions should complement checklist implementation to support long-term adherence to safety protocols [33].

Regarding double-checking, while the participants acknowledged its relevance, they emphasized that staff shortages rendered this strategy impractical. Double-checking has shown potential in reducing medication errors in specific situations, but it remains fallible if performed superficially or under time pressure [34,35]. Furthermore, evidence on the effectiveness of double-checking remains inconclusive, with some studies finding no significant reduction in error rates when this strategy is implemented [36,37]. Despite these mixed findings, participants recognized the safety benefits of double-checking but cited the lack of time and staff as significant barriers to its consistent use, particularly in high-demand settings like ICUs.

The participants also noted that discussion groups and simulated practice sessions on medication preparation and administration should be sporadic rather than routine. The literature supports this by advocating for evidence-based educational programs tailored to medication management, complemented by clinical training and periodic reminders [38]. A systematic approach is required to reduce organizational vulnerability to errors, emphasizing the importance of monitoring, analyzing, and implementing preventive measures [39].

A notable finding from the discussion was the underutilization of the existing error reporting system in the ICU. Despite recognizing that medication errors occur, participants reported that errors are rarely documented. This lack of reporting is primarily attributed to an inadequate knowledge of the reporting process and a fear of punitive consequences. Research corroborates that most medication errors go unreported, but reporting rates can increase significantly following educational interventions [38]. This highlights the need for practical training sessions and initiatives to foster a non-punitive culture of reporting.

### 4.1. Recommendations for Nursing

Implementing interventions to minimize medication errors in intensive care units is essential for advancing nursing practice, enhancing patient safety, and improving the quality of care. These interventions also have the potential to promote the professional development of nurses while supporting nursing managers in optimizing operational efficiency. Furthermore, they contribute to creating a safer and more collaborative work environment.

One critical recommendation is the promotion of a safety culture within healthcare settings. Establishing a positive and open work environment, where staff feel encouraged to communicate and collaborate without fear of blame or punitive actions, is fundamental. A strong safety culture fosters trust among team members, enabling the identification and resolution of issues that may contribute to medication errors. Nurses must be empowered to report errors, reflect on their practice, and participate in developing solutions to enhance care quality.

In addition, robust error reporting tools are crucial for identifying system weaknesses and implementing continuous improvement programs. Such systems should prioritize anonymity and non-discrimination, ensuring that nurses feel secure in reporting incidents. Educating staff on how to effectively use these tools, coupled with feedback sessions that demonstrate how reported errors lead to actionable improvements, is vital to fostering their adoption.

Ongoing research is also necessary to address persistent medication errors. Despite existing strategies, the complexity of ICU environments and nursing tasks calls for continued efforts to refine and test innovative approaches. Future studies should aim to explore the underlying causes of medication errors and their broader impacts, enabling the development of targeted interventions that address these root issues.

Finally, advancing nursing education is a cornerstone for reducing medication errors. From the early stages of training, nursing curricula should incorporate evidence-based strategies, technological tools, and simulations to prepare future nurses for the challenges of medication management. Educational programs must also adapt to the dynamic nature of healthcare, integrating updates from current research and clinical guidelines to ensure that nursing practice evolves alongside emerging knowledge and technologies.

These recommendations provide a foundation for future research and practice, offering a comprehensive approach to improving patient safety while advancing the nursing profession.

### 4.2. Study Limitations

This study offers valuable insights into strategies for reducing medication errors in ICUs, but certain limitations should be noted.

A major limitation is the intervention’s context-specific nature. While the methodology for designing and validating the intervention can be replicated, the findings may not fully apply to all ICUs due to organizational and cultural differences, requiring further adaptation for effectiveness.

Additionally, a scoping review informed the evidence base used to develop the intervention, but the available research on complex nursing interventions remains limited. This gap underscores the need for further studies in patient safety and evidence-based programs to reduce nursing errors.

Recognizing these limitations is crucial for interpreting the findings and guiding future research. Broader participant inclusion, cross-context validation, and expanded studies will improve the generalizability and effectiveness of such interventions.

## 5. Conclusions

Implementing complex interventions to mitigate errors, such as medication errors, can significantly enhance patient safety by improving the quality of care delivery.

The intervention developed in this study is structured around four key components—educational interventions, verification and safety methods, organizational and functional modifications, and an error reporting system. These components were meticulously designed to leverage existing ICU resources while involving competent professionals to ensure the feasibility and appropriateness of the strategies. This intervention adopts an organizational-level approach, employing a multifaceted strategy that includes healthcare professional training, the provision of educational materials, collaborative care practices, and functional improvements. These elements address critical vulnerabilities and enhance patient safety and care quality.

The critical analysis provided by the focus group participants underscored the importance of tailoring interventions, particularly for less experienced nurses, by integrating discussion sessions on safety, simulated practice, and checklists. Additionally, the underutilization of the error reporting system revealed a significant gap in the organizational culture of the studied ICU. This highlights the necessity for educational interventions that promote a non-punitive reporting culture and enhance awareness of the error reporting processes.

While this study concludes with constructing a complex intervention, its relevance lies in its potential implementation within the studied context. Such implementation is expected to significantly reduce errors and foster greater awareness of the need to cultivate a safety and error reporting culture.

Beyond its practical implications, this study meaningfully contributes to the broader discussion of healthcare errors, offering a platform for reflection and learning for professionals and stakeholders. It emphasizes the importance of reinforcing safety practices, fostering a culture of continuous improvement, and prioritizing patient-centered care. The findings and proposed intervention provide a foundation for future research and practical applications, supporting the ongoing pursuit of excellence in healthcare delivery.

## Figures and Tables

**Table 1:** Components and complex intervention plan.

Intervention Components	Intervention Plan and Activities
Educational intervention (knowledge and training)	- Create groups and moments of discussion on the safety of the medication process. - Post posters, distribute leaflets and information booklets, and disseminate educational videos and memory aids on aspects notified, such as errors, checklists, and protocols. - Make PowerPoint presentations (in discussion and feedback sessions on errors and as a pedagogical formative tool). - Promote moments of simulated practice on the preparation and administration of medication.
Verification and safety methods (procedure-related factors)	- Developing and using protocols and operating instructions. - Create and use checklists for drug administration. - Check laboratory values before administration. If in doubt, contact the prescriber. - Do not interrupt the preparation and administration of medication (create non-interruption mechanisms unless the information is relevant). - Have two nurses double-check the preparation of the medication. - Monitor vital signs before and after administering the IV medication.
Organizational and functional changes (organizational constraints and system-related factors)	- Establish different colors, designs, and labels to differentiate between different containers of medicines with similar appearances. - Store medicines with similar labels in different places. - Manage medicines using barcodes and an electronic documentation system rather than paper documentation systems.
Error notification system	- Implement an anonymous error reporting system. - Create reports on medication-related events that derive from the reporting process and use them as an information tool in feedback sessions for professionals and discussion groups.

**Table 2:** Characterization of the participants in the focus group.

Participant (P)	Age	Gender	Length of Professional Career (in Years)	Time in ICU (in Years)	Academic Qualifications (Highest Academic Degree)	Other Qualifications
P1	45	Male	25	25	Master	-Postgraduate Degree in Management -Postgraduate Degree in Nursing Information Systems
P2	37	Male	13	9	Master	
P3	51	Male	29	20	Master	-Postgraduate Degree in Health Organization Management
P4	46	Female	20	7	Graduate	-Postgraduate Degree in Health Organization Management
P5	35	Female	13	9	Master	-Postgraduate Degree in Critical Care -Postgraduate Degree in Neuropsychology
P6	39	Male	16	12	Master	

**Table 3:** Components and plan of the complex intervention validated in the context of the ICU under study.

Intervention Components	Intervention Plan and Activities
Educational intervention (knowledge and training)	- Hold discussions to discuss strategies and define intervention plans. - Create discussion groups with less experienced nurses who are just starting, to integrate them and discuss how to improve their difficulties. - Display posters and checklists in the medication preparation area. - Film moments of medication preparation for later viewing and analyzing points for improvement. - Use PowerPoint to share data resulting from monitoring the indicators associated with the medication management process and to provide pharmacology training. - Promote moments of simulated practice on medication preparation and administration for less experienced nurses who are in the process of joining the service.
Verification and safety methods (procedure-related factors)	- Develop and use protocols and operational instructions for medication management for specific medications. - Create and use checklists for medication administration. - Do not interrupt medication preparation and administration (create non-interruption mechanisms, except if the information is transmitted for relevant purposes).
Organizational and functional changes (organizational constraints and system-related factors)	- Establish distinct colors, designs, and labels to differentiate containers of medicines with similar appearances, particularly those of greater criticality. - Improve the electronic management system in place in the service by implementing a method of printing labels in a different color for high-risk or highly critical medicines.
Error notification system	- Provide practical training on the error reporting process in the existing notification platform. - Create reports on medication-related events arising from the notification process and use them to identify areas for improvement.

## Data Availability

The original contributions presented in this study are included in the article. Further inquiries can be directed to the corresponding author.

## Appendix A. Focus Group Interview Guide

**Interview Guide**	
**Study title:** Preventing Medication Errors in Intensive Care: A Case Study	
Focus Group Date: ___/___/_______	Start: ______ End: ______
**Legitimation of the Interview**	
- Identification/introduction of the interviewer - Presenting the topic, research question, and study objectives - Clarifying procedures and addressing any questions - Informing participants about the recording of the interview - Ensuring confidentiality and privacy matters are addressed - Confirming the signature of the Informed Consent Form - Expressing gratitude for participation and collaboration in the study	
**Questions Guiding the Focus Group**	
Each question is presented individually, allowing participants to share their perspectives on each strategy.	
1. What is your opinion on the suitability of the following educational strategies to minimize medication errors? 1.1. Creating discussion groups and dedicated moments to address the safety of the medication process 1.2. Posting posters, distributing pamphlets and informational leaflets, sharing educational videos, memory aids on reported errors, checklists, protocols, and similar materials 1.3. Conducting PowerPoint presentations (during discussion sessions and feedback on errors, as well as using them as a pedagogical training tool) 1.4. Promoting simulation-based practice sessions on medication preparation and administration	
2. Regarding verification and safety methods, what is your opinion on the suitability of the following strategies to minimize medication errors? 2.1. Developing and implementing protocols and/or operational instructions 2.2. Creating and using checklists for medication administration 2.3. Verifying laboratory values before administration and, if in doubt, consulting the prescriber 2.4. Avoiding interruptions during medication preparation and administration (establishing mechanisms to prevent interruptions unless the information is critical) 2.5. Conducting double-checks by two nurses during medication preparation 2.6. Monitoring vital signs before and after IV medication administration	
3. Considering the component of organizational and functional modifications, do you find the following strategies suitable and feasible in your ICU? 3.1. Establishing distinct colors, designs, and labels to differentiate containers of medications with similar appearances 3.2. Storing medications with similar labels in separate locations 3.3. Managing medications using barcode systems and transitioning to electronic documentation systems instead of paper-based documentation	
4. The error reporting system was identified as a key intervention component. Do you consider the following strategies feasible? 4.1. Implementing an anonymous error reporting system 4.2. Creating reports on medication-related events derived from the reporting process and using them as informational tools during feedback sessions and discussion groups	
**Focus Group Closure**	
- Asking about relevant aspects to add - Offering availability for additional clarifications - Expressing gratitude for participation and collaboration in the study	
